# Exogenous 8-hydroxydeoxyguanosine attenuates doxorubicin-induced cardiotoxicity by decreasing pyroptosis in H9c2 cardiomyocytes

**DOI:** 10.1186/s12860-022-00454-1

**Published:** 2022-12-14

**Authors:** Soyoung Hwang, Se-Hee Kim, Kwai Han Yoo, Myung-Hee Chung, Jin Woo Lee, Kuk Hui Son

**Affiliations:** 1 Department of Thoracic and Cardiovascular Surgery, Gachon University Gil Medical Center, College of Medicine, Gachon University, 21, Namdong-daero 774 beon-gil, Namdong-gu, Incheon, Republic of Korea; 2 Gachon Medical Research Institute, Gachon University Gil Medical Center, College of Medicine, Gachon University, 38-13, Dokjeom-ro 3 beon-gil, Namdong-gu, Incheon, Republic of Korea; 3 Division of Hematology, Department of Internal Medicine, Gachon University Gil Medical Center, Gachon University College of Medicine, Incheon, Korea; 4 Lee Gil Ya Cancer and Diabetes Institute, Gachon University, 155, Gaetbeol-ro, Yeonsu-ku, Incheon, Republic of Korea; 5 Department of Molecular Medicine, College of Medicine, Gachon University, 155, Gaetbeol-ro, Yeonsu-ku, Incheon, Republic of Korea; 6 Department of Health Sciences and Technology, GAIHST, Gachon University, 155, Gaetbeol-ro, Yeonsu-ku, Incheon, Republic of Korea

**Keywords:** Exogenous 8-hydroxydeoxyguanosine, Doxorubicin, Cardiotoxicity, Pyroptosis

## Abstract

Doxorubicin (DOX), which is widely used in cancer treatment, can induce cardiomyopathy. One of the main mechanisms whereby DOX induces cardiotoxicity involves pyroptosis through the NLR family pyrin domain containing 3 (NLRP3) inflammasome and gasdermin D (GSDMD). Increased NAPDH oxidase (NOX) and oxidative stress trigger pyroptosis. Exogenous 8-hydroxydeoxyguanosine (8-OHdG) decreases reactive oxygen species (ROS) production by inactivating NOX. Here, we examined whether 8-OHdG treatment can attenuate DOX-induced pyroptosis in H9c2 cardiomyocytes. Exposure to DOX increased the peroxidative glutathione redox status and NOX1/2/4, toll-like receptor (TLR)2/4, and nuclear factor kappa-light-chain-enhancer of activated B cells (NF-κB) expression, while an additional 8-OHdG treatment attenuated these effects. Furthermore, DOX induced higher expression of NLRP3 inflammasome components, including NLRP3, apoptosis-associated speck-like protein containing a c-terminal caspase recruitment domain (ASC), and pro-caspase-1. Moreover, it increased caspase-1 activity, a marker of pyroptosis, and interleukin (IL)-1β expression. All these effects were attenuated by 8-OHdG treatment. In addition, the expression of the cardiotoxicity markers, atrial natriuretic peptide (ANP) and brain natriuretic peptide (BNP) was increased by DOX, whereas the increase of ANP and BNP induced by DOX treatment was reversed by 8-OHdG. In conclusion, exogenous 8-OHdG attenuated DOX-induced pyroptosis by decreasing the expression of NOX1/2/3, TLR2/4, and NF-κB. Thus, 8-OHdG may attenuate DOX-induced cardiotoxicity through the inhibition of pyroptosis.

**Supplementary Information:**

The online version contains supplementary material available at 10.1186/s12860-022-00454-1.

## Introduction

Doxorubicin (DOX) is widely used as a treatment for numerous cancers, such as breast cancer, soft tissue sarcomas, lymphomas, and leukemia. Despite its effectiveness, DOX can induce serious complications such as cumulative cardiotoxicity [1, 2]. It has been known that DOX-induced cardiotoxicity is mainly caused by cumulative effect, thus it has been recommended that accumulation doses should not exceed 500 mg/m^2^ [3]. However, resent studies showed that Dox-induced cardiotoxicity could develop even by single administration of DOX [4, 5]. DOX-induced cardiotoxicity leads to irreversible myocardial injury or congestive heart failure [6]. The pathophysiology of DOX-induced cardiotoxicity has not been fully revealed; however, oxidative stress, apoptosis, inflammation, and impaired regulation of autophagy may be involved in its development [7–10]. Moreover, NLR family pyrin domain containing 3 (NLRP3) inflammasome formation, which leads to gasdermin D (GSDMD)-dependent pyroptosis, has recently been identified as one of the main mechanisms whereby DOX induces cardiotoxicity [11].

Damage-associated molecular patterns (DAMPs) are released from dying cells and are recognized by NOD-like receptors such as NLRP3 [12]. The NLRP3 inflammasome consists of the sensor molecule NLRP3, inflammasome adaptor apoptosis-associated speck-like protein containing a c-terminal caspase recruitment domain (ASC), and pro-caspase-1 [13, 14]. NLRP3 inflammasome activation results in caspase-1 activation through the cleavage of pro-caspase-1 [13, 14]. In turn, the activation of caspase-1 leads to the maturation of IL-1β and IL-18 through the cleavage of pro-IL-1β and pro-IL-18 [15–17]. Moreover, activated caspase-1 cleaves GSDMD, and the resulting N-terminal cleavage product (GSDMD-NT) binds to the plasma membrane to form pores [15, 16]. Through these pores, IL-1β and IL-18 are released into the extracellular space, which aggravates inflammation [17–19]. Furthermore, formation of pores in the cell membrane, and the release of inflammatory factors cause cell swelling, membrane rupture, and pyroptosis [15, 20–22]. Pyroptosis involves in development of various diseases such as diabetic cardiomyopathy, myocardial infarction, and diabetic renal endothelial cell damage [23–25]. Furthermore, suppressing pyroptosis by sodium-glucose co-transporter-2 (SGLT-2) inhibitors, proprotein convertase subtilisin/Kexin type 9 (PCSK9) inhibitor, or sodium butyrate is considered to have possibility as therapeutics for those diseases which are associated with pyroptosis [23–25].

Tumor necrosis factor (TNF)-α expression is increased in DOX-induced cardiotoxicity [26, 27], and this is also known to lead to pyroptosis [28]. Toll-like receptors (TLRs) are highly expressed in cardiomyocytes and related to DOX-induced cardiotoxicity [28]. In DOX-induced cardiotoxicity, the activation of TLR-4 and TLR-2 leads to the upregulation of nuclear factor kappa-light-chain-enhancer of activated B cells (NF-κB), which results in increased expression of various pro-inflammatory cytokines, including TNF-α and IL-6 [29, 30]. TLR/NF-κB signaling increases NLRP3 binding to ASC [31]. Furthermore, increased oxidative stress is involved in DOX-induced cardiotoxicity [32]. Oxidative stress is the result of both an increased production of reactive oxygen species (ROS) and a decreased production of endogenous antioxidants such as catalase (CAT), superoxide dismutase (SOD), and glutathione (GSH) in the cellular system [32, 33]. NADPH oxidase (NOX)1 and NOX4 are also involved in the activation of the NLRP3 inflammasome through the increase in ROS production during DOX-induced cardiotoxicity [34]. NOX2 is also involved in the activation of the NLRP3 inflammasome in brain injuries [35]. In addition, TLR4 promotes NOX4-mediated ROS production [36], and TLR2 stimulates NOX1 and NOX2 to generate ROS [28, 37]. Increased expression of TLR2 also lead to pyroptosis via TLR2/Myd88/ NF-κB pathway [38].

Endogenous 8-OHdG is frequently used as an indicator of the oxidative DNA damage induced by ROS, as it is an oxidized nucleoside of DNA [39]. Paradoxically, exogenous 8-OHdG is known to decrease ROS production by inhibiting the Ras-related C3 botulinum toxin substrate 1 (Rac1) and NOX complexes [39]. Moreover, exogenous 8-OHdG decreases pro-inflammatory cytokine production, including IL-1β, TNF-α, and IL-6, in the adipose tissue by inhibiting the NOX complex [40]. Furthermore, exogenous 8-OHdG downregulates the NF-κB pathway in the gastrointestinal tract [39].

Here, we examined whether exogenous 8-OHdG can attenuate DOX-induced pyroptosis in H9c2 cardiomyocytes. We hypothesized that treatment with exogenous 8-OHdG would lead to the downregulation of NOX, and NF-κB expression, and decreased TNF-α production by decreasing Rac1, eventually leading to the attenuation of DOX-induced pyroptosis.

## Materials and methods

### Cell lines and reagents

H9c2 cells were obtained from ATCC (Manassas, VA, USA) and grown in Dulbecco’s modified Eagle’s medium (DMEM) (Welgene, Daegu, South Korea) with 10% fetal bovine serum (FBS, Welgene) and 1% penicillin–streptomycin (Welgene). 8-Hydroxydeoxyguanosine (8-OHdG), was provided by Professor Myung-Hee Chung. DOX was obtained from Seleckchem (Huston, TX, USA).

### Cell viability assay

1 × 10^4^ cells were plated in 96-well culture plates, treated with DOX or 8-OHdG, and incubated for 24, 48, or 72 h at 37 °C. Then, the cells were incubated in medium containing 0.5 mg/mL 3-(4,5-dimethylthiazol-2-yl)-2,5-diphenyltetrazolium bromide (MTT) (#M2128, Sigma-Aldrich, St. Louis, MO, USA) for 1 h at 37 °C. Dimethyl sulfoxide (DMSO) was added at the end of the reaction to dissolve the formazan crystals from viable cells. The absorbance was measured at 560 nm with a microplate reader (Emax, Molecular Devices, San Jose, CA, USA).

### Lactated dehydrogenase assay

2 × 10^4^ cells were seeded in 96-well plates and treated with DOX and 8-OHdG in culture medium containing 1% FBS for 24–48 h at 37 °C. Cell-free culture supernatants were mixed with lactate dehydrogenase (LDH) reaction mixture (#MK401, Takara Bio Inc., Otsu, Shiga, Japan) and incubated for 30 m at room temperature. The absorbance was measured at 490 nm.

### Rac1 activation assay

The Rac1 activity was measured using Rac1 activation assay kit (Cell Biolabs, San Diego, CA, USA) according to the manufacturer’s protocol. Briefly, for immunoprecipitation, 200 μg proteins were incubated in assay buffer containing p21-activated protein kinase (PAK) p21-binding domain (PBD) agarose beads at 4℃ for overnight. After washing with 1X assay buffer, pull-down supernatants were separated in 12% SDS–PAGE gels. Primary antibodies to Rac1 (1:1000) (Cell Biolabs) and β-actin (1:3000) (Santa Cruz Biotechnology) were used. Images were taken with an Amersham Imager 600 (GE healthcare, Chicago, IL, USA).

### Quantitative real time PCR

Total RNA was isolated from H9c2 cells (3 × 10^5^ cells/60 mm dish) using RNAiso plus (9108, Takara Bio) and a Qiagen RNeasy Mini Kit (74104, Qiagen, Hilden, Germany). RNA quantitation was performed using NanoDrop-2000 (Thermo Fisher Scientific, Waltham, MA, USA). Then, cDNA synthesis was performed using a PrimeScript First Strand cDNA Synthesis Kit (#6110A, Takara Bio). Real-time PCR was performed on a CFX96 real-time system (Bio-Rad, Hercules, CA, USA) using SYBR Green I Universal PCR Master Mix (Takara Bio) and primers (Bioneer, Daejeon, South Korea) in reactions with the following conditions: 95 °C for 10 min, followed by 40 cycles of 95 °C for 15 s, and 60 °C for 1 min. GAPDH was used as a reference gene. Relative gene expression presents the data of the gene of interest relative to internal control gene using the comparative *C*_T_ method also referred to as the 2^−△△*C*T^ method. Primer (Bioneer, Daejeon, South Korea) sequences for rat TNF-α (GeneBank Accession no. AB553578.1), NLRP3 (GeneBank Accession no. XM_039085397.1), atrial natriuretic peptide (ANP) (GeneBank Accession no. M27498.1), brain natriuretic peptide (BNP) (GeneBank Accession no. XM_032887581.1), GATA binding protein 4 (GATA4) (GeneBank Accession no. XM_032917655.1), GATA binding protein 6 (GATA6) (GeneBank Accession no. NM_019185.2), TLR-2 (GeneBank Accession no. NM_198769.2), TLR-4 (GeneBank Accession no. NM_019178.2), and GAPDH (GeneBank Accession no. NM_017008) are listed in Table 1.

**Table 1:** Nucleotide sequences of qRT-PCR primers

**PCR targets**	**Forward primer (5’–3’)**	**Reverse primer (5’–3’)**
TNF-α	CATCCGTTCTCTACCCAGCC	AATTCTGAGCCCGGAGTTGG
IL-1β	TGCTGTCTGACCCATGTGAG	GTCGTTGCTTGTCTCTCCTTG
NLRP3	GTGGAGATCCTAGGTTTCTCTG	CAGGATCTCATTCTCTTGGATC
ASC	CTCTGTATGGCAATGTGCTGAC	GAACAAGTTCTTGCAGGTCAG
Caspase-1	GAGCTGATGTTGACCTCAGAG	CTGTCAGAAGTCTTGTGCTCTG
ANP	AAATCCCGTATACAGTGCGG	GGAGGCATGACCTCATCTTC
BNP	CCAGAACAATCCACGATGC	TCGAAGTCTCTCCTGGATCC
GATA4	CCCCAATCTCGATATGTTTGATGAC	GGGCCGGTTGATACCATTCA
GATA6	TGAACGGGACGTACCACCACCACC	ACAGTTCACGCACTCGCGGCTCTC
TLR2	GGCCACAGGACTCAAGAGCA	AGAGGCCTATCACAGCCATCAAG
TLR4	GGACTCTGCCCTGCCACCATTTA	CTTGTGCCCTGTGAGGTCGTTGA
GAPDH	GGCTCTCTGCTCCTCCCTGTTCTA	TGCCGTTGAACTTGCCGTGGG

*TNF* Tumor necrosis factor, *IL* Interleukin, *NLRP3* NLR family pyrin domain containing 3, *ASC* Apoptosis-associated speck-like protein containing a c-terminal caspase recruitment domain, *ANP* Atrial natriuretic peptide, *BNP* Brain natriuretic peptide, *TLR* Toll-like receptor, *GAPDH* Glyceraldehyde 3-phosphate dehydrogenase

### Western blot analysis

Cells (3 × 10^5^ cells/60 mm dish) were lysed in RIPA buffer (20 mM Tris–HCl (pH 7.5), 150 mM NaCl, 1 mM EGTA, 2.5 mM sodium pyrophosphate, 1 mM β-glycerophosphate, 1 mM sodium orthovanadate, 1 μg/ml leupeptin, 1% sodium deoxycholate, 1% NP-40) (#9806, Cell signaling technology, Danvers, MA, USA). 10–20 μg proteins were separated in 8–12% SDS–PAGE gels and transferred onto PVDF membranes. After blocking with 5% skim milk, the membranes were incubated with primary antibodies at 4℃ for overnight. Primary antibodies for ASC (1:1000) (GeneBank Accession no. NM_172322.1) (sc-271054, Santa Cruz, Dallas, TX, USA), caspase-1 (1:1000) (GeneBank Accession no. NM_012762.3) (NBP1-45433, Novus biologicals, Denver, CO, USA), caspase 3 (1:1000) (#9661S, Cell signaling technology), IL-1β (1:1000) (GeneBank Accession no. XM_032902343.1) (NB600-633, Novus biologicals), NOX1 (1:1000) (GeneBank Accession no. NM_053683.2) (NBP1-31546, Novus biologicals), NOX2 (1:1000) (GeneBank Accession no. NM_023965.1) (NBP2-41291, Novus biologicals), NOX4 (1:1000) (GeneBank Accession no. NM_053524.1) (NB110-58851, Novus biologicals), NF-κB p65 (1:2000) (GeneBank Accession no. AJ002424.2) (#8242, Cell signaling technology), Phospho-NF-κB p65 (1:1000) (#3033, Cell signaling technology), GSDMD-NT (1:1000) (ER1901-37, HUAbio, Boston, MA), and β-actin (1:3000) (SC-47778, Santa Cruz) were used. Images were taken with an Amersham Imager 600 (GE healthcare, Chicago, IL, USA). Band intensities were normalized to β-actin using ImageJ software (NIH, Bethesda, MD, USA).

### Pyroptosis assay

3 × 10^5^ cells were plated in 60 mm dishes and treated with DOX and 8-OHdG for 24 h. Caspase-1 activation was examined with a pyroptosis/caspase-1 assay kit (MBS258046, Mybiosource, San Diego, CA, USA) as per the manufacturer’s instructions. First, cells were incubated in medium containing fluorochrome-labeled inhibitors of caspases (FLICA) for 4 h at 37 °C in the dark. Then, the cells were washed and transferred to black microtiter plates to measure fluorescence intensity (excitation wavelength, 480 nm; emission wavelength, 515 nm) with a microplate reader (Molecular Devices). Then, cells were fixed with fixation buffer for 2 min, washed with Dulbecco’s phosphate-buffered saline (DPBS), placed on a glass slide to which 50 μL antifade mounting medium (H-1000–10, Vector Laboratories Inc., Burlingame, CA, USA) was applied, and covered with 25 mm cover glasses. Images were taken with an LSM 700 Zeiss confocal microscope (Carl Zeiss, Oberkochen, Germany) and analyzed using ZEN software (Carl Zeiss). The experiment was performed three times.

### Glutathione assay

Cells were plated in 100 mm dishes and treated with DOX and 8-OHdG for 24 h. A glutathione assay kit (Abcam, Cambridge, UK) was used as per the manufacturer’s instructions. Briefly, cell suspension from lysed 10^6^ cells using lysis buffer was incubated in medium containing thiol or GSSG probe at room temperature for 10–60 m. Then, we measured concentrations of GSH and total glutathione (GSH + GSSG) at Ex/Em = 490/520 nm using a fluorescence microplate reader (Thermo Scientific, Waltham, MA, USA). The GSH/GSSG ratio was calculated from concentrations of GSH and GSSG.

### Statistical analysis

All experiments were repeated more than three times, and the average values are presented unless otherwise stated. Data are presented as mean ± standard deviation. The statistical significance of the results was determined with Prism® software (GraphPad, San Diego, CA, USA) using one-way ANOVA and post-hoc Dunnett’s test. Significance was defined as *P < 0.05, **P < 0.01, and *** *P* < 0.001 in all experiments.

## Results

### DOX decreases H9c2 cell viability in a dose-dependent manner

First, we performed preliminary experiments to determine the appropriate toxic concentration of DOX and the effective concentration of 8-OHdG. Cells were treated with 0.1, 1, 5, 10, and 20 μM DOX or 25, 50, 100, 250, 500, and 1000 μg/mL 8-OHdG for 24, 48, and 72 h. Then, cell viability was evaluated with the MTT assay.

**Fig. 1:**
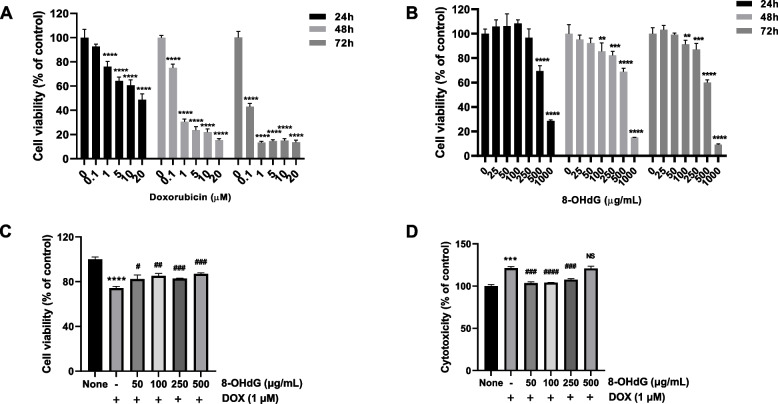
Viability and cytotoxicity of doxorubicin- and 8-hydroxydeoxyguanosine-treated H9c2 cells. An MTT assay was used to examine H9c2 cell viability after treatment with various concentrations of DOX (**A**) and 8-OHdG (**B**) for 24, 48, and 72 h. **C**, After H9c2 cells were treated with DOX (1 μM) and 8-OHdG (50, 100, 250, and 500 μg/mL) for 24 h, cell proliferation and cytotoxicity were measured using MTT (**C**) and LDH (**D**) assays, respectively. DOX, doxorubicin; 8-OHdG, 8-hydroxydeoxyguanosine. ^**^P < 0.01, ^***^P < 0.001, and ^****^P < 0.0001 versus control; ^#^*P* < 0.05, ^##^*P* < 0.01, ^###^*P* < 0.001, and ^####^*P* < 0.001 versus DOX

The results showed that cell viability gradually decreased as the concentration of DOX increased (Fig. 1A). The IC_50_ values of DOX over the study period were 20.6 μM (24 h), 0.4778 μM (48 h), and 0.02895 μM (72 h). After 24 h culture, the viability of 8-OHdG-treated H2c9 cells did not significantly differ from that of the control when the 8-OHdG concentration ranged from 0 to 250 μg/mL; however, cell viability was significantly decreased when the concentration was ≥ 500 μg/mL (Fig. 1B). After 48 and 72 h culture, the viability of 8-OHdG-treated H2c9 cells did not significantly differ from that of the control when the 8-OHdG concentration ranged from 0 to 50 μg/mL (Fig. 1B). The IC_50_ values of 8-OHdG were 699.4 μg/mL (24 h), 595.5 μg/mL (48 h), and 544.4 μg/mL (72 h). To evaluate the effect on pyroptosis, we used 1 μM DOX as in previous reports [41]. In addition, we examined cell proliferation and cytotoxicity in 1 μM DOX-treated cells by treating them with various concentrations (50, 100, 250, and 500 μg/mL) of 8-OHdG. 50, 100, and 250 μg/mL of 8-OHdG augmented the proliferation of DOX-treated H9c2 cells and conversely alleviated the cytotoxicity of these cells (Fig. 1C and D). Therefore, we treated these H9c2 cells with 1 μM of DOX and 100 and 250 μg/mL of 8-OHdG for following investigation.

### Exogenous 8-OHdG decreases the expression of cardiotoxicity-related markers in DOX-treated H9c2 cells

**Fig. 2:**
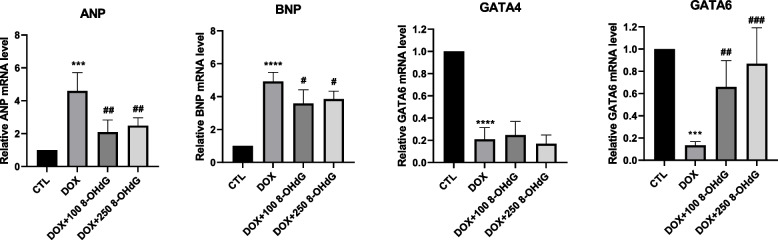
Exogenous 8-OHdG decreases the expression of cardiotoxicity-related markers in DOX-treated H9c2 cells. H9c2 cells were treated with 1 μM DOX and 100 or 250 μg/mL 8-OHdG for 24 h. Quantitative real-time PCR for ANP, BNP, GATA4, and GATA6 in DOX and 8-OHdG treated H9c2 cells. CTL, control; DOX, doxorubicin; 8-OHdG, 8-hydroxydeoxyguanosine; ANP, atrial natriuretic peptide; BNP, brain natriuretic peptide. ^***^P < 0.001 and ^****^P < 0.0001 versus control; ^#^*P* < 0.05, ^##^*P* < 0.01, and ^###^*P* < 0.001 versus DOX

Cytotoxicity of DOX-treated H9c2 cells was reduced by 8-OHdG treatment (Fig. 1). Thus, we tested whether exogenous 8-OHdG affects the expression of cardiac hypertrophy markers, ANP and BNP in DOX-treated H9c2 cells. DOX increased ANP and BNP mRNA levels by 258–479% and 337–428%, respectively, compared to those in control cells, whereas the addition of 8-OHdG reduced their expression levels by 18.4–78.1% and 16.4–49.1% in DOX-treated cells (Fig. 2). Therefore, 8-OHdG mitigates DOX-induced cardiac toxicity in H9c2 cells.

GATA4 and GATA6 are transcription factors involved in cell survival. We assessed the effect of DOX and 8-OHdG on the expression of GATA4 and 6 in cardiomyocytes. As shown in Fig. 2, DOX exposure reduced the mRNA levels of GATA4 and 6 by 68–90% and 83.4–91% compared to those in control cells. However, additional treatment with 8-OHdG only recovered GATA6 expression, but not GATA4 expression, in DOX-treated H9c2 cells. These findings suggest that 8-OHdG may attenuate DOX-induced cardiotoxicity in H9c2 cells, and that GATA6 may be involved.

### Exogenous 8-OHdG decreases NOX1/2/4 expression and NF-κB phosphorylation, and increases the GSH/GSSG ratio in DOX-treated H9c2 cells

Given that 8-OHdG is involved in oxidative stress via Rac1, we examined the regulatory effects of DOX and 8-OHdG on expression of NOX1/2/4 and the Rac1 activity in H9c2 cells.

**Fig. 3:**
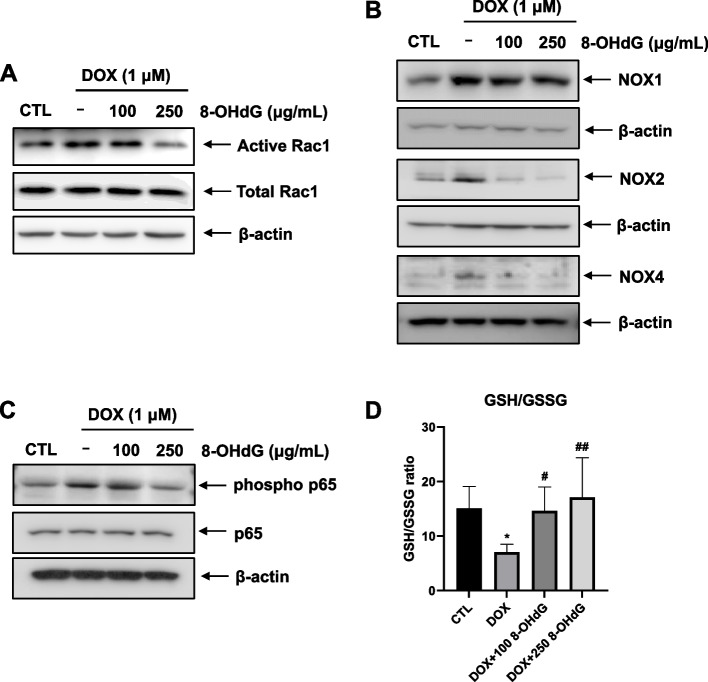
Exogenous 8-OHdG decreases NOX1/2/4 expression and NF-κB phosphorylation, and increases the reduced glutathione/oxidized glutathione ratio in DOX-treated H9c2 cells. **A** Rac1 activation assay. After H9c2 cells were treated with DOX (1 μM) and 8-OHdG (100 or 250 μg/mL) for 1 h, cell lysates were precipitated by p21-activated protein kinase (PAK) p21-binding domain (PBD) agarose beads and immunoblotted by Rac1 specific monoclonal antibody. **B**–**D** H9c2 cells were treated with 1 μM DOX and 100 μg/mL or 250 μg/mL 8-OHdG for 24 h. **B**–**C** Western blot analysis of the NOX1/2/4, p65, and phosphor-p65 protein levels of in DOX- and 8-OHdG-treated H9c2 cells. **D** GSH/GSSG ratio was determined using a glutathione assay kit. CTL, control; DOX, doxorubicin; 8-OHdG, 8-hydroxydeoxyguanosine; NOX, NADPH oxidase; GSH/GSSG, reduced glutathione/oxidized glutathione ratio. ^*^P < 0.05 versus control; ^#^*P* < 0.05 and ^##^*P* < 0.01 versus DOX-treated groups

We performed the Rac1 activation assay using immunoprecipitation and found that 250 μg/mL 8-OHdG decreased the rat Rac1 activity in DOX-exposed H9c2 cells (Fig. 3A). Additionally, when cells were treated with both DOX and 8-OHdG, the protein levels of NOX1, 2, and 4 were decreased compared to DOX-treated cells (Fig. 3B). However, 8-OHdG suppressed the expression of NOX2 and NOX4 more than that of NOX1 in DOX-treated cells. In addition, exogenous 8-OHdG repressed the phosphorylation of p65 compared to the levels in DOX-treated H9c2 cells (Fig. 3C). These findings suggest that 8-OHdG may mitigate DOX-induced NOX1/2/4 upregulation through the inactivation of p65 and Rac1.

Oxidative stress is the result of both the increased production of ROS and decreased content of endogenous antioxidants such as CAT, SOD, and GSH in the cellular system [32, 33]. Thus, we examined whether 8-OHdG treatment affects the GSH/GSSG ratio in DOX-treated H9c2 cells. Exogenous 8-OHdG recovered the ratio of GSH/GSSG by 13.8–389% in DOX-treated H92C cells (Fig. 3D). Collectively, the results demonstrated that 8-OHdG treatment decreases NOX1/2/4 expression and increases the GSH/GSSG ratio in DOX-treated H9c2 cells, suggesting that 8-OHdG may alleviate DOX-induced oxidative stress in cardiomyocytes.

### Exogenous 8-OHdG decreases the expression of NLRP3 inflammasome components and caspase-1 in DOX-treated H9c2 cells

**Fig. 4:**
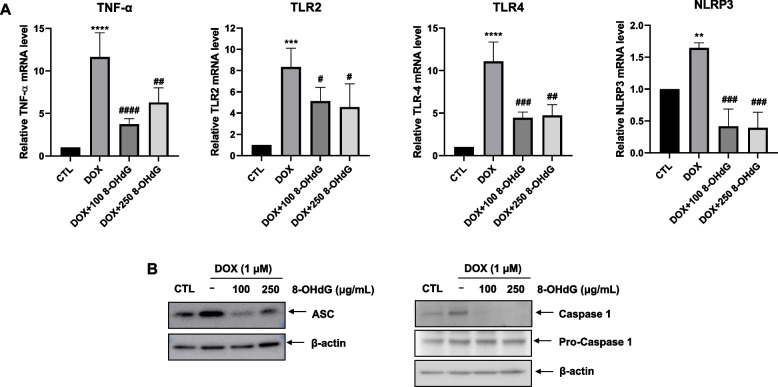
Exogenous 8-OHdG decreases the expression of NLRP3, ASC, and caspase-1 in DOX-treated H9c2 cells. **A-B** H9c2 cells were treated with 1 μM DOX and 100 μg/mL or 250 μg/mL 8-OHdG for 24 h. **A** Quantitative real-time PCR for TNF-α, TLR2, TLR4, and NLRP3 in DOX- and 8-OHdG-treated H9c2 cells. **B** Western blot analysis ASC and caspase-1 protein levels in DOX- and 8-OHdG-treated H9c2 cells. CTL, control; DOX, doxorubicin; 8-OHdG, 8-hydroxydeoxyguanosine; TNF, tumor necrosis factor; TLR, toll-like receptor; NLRP, NLR family pyrin domain containing; ASC, apoptosis-associated speck-like protein containing a CARD c-terminal caspase recruitment domain. ^**^P < 0.01 and ^****^P < 0.0001 versus control; ^#^*P* < 0.05, ^##^*P* < 0.01, ^###^*P* < 0.001, and ^####^*P* < 0.0001 versus DOX-treated groups

DOX-induced cardiotoxicity increases TNF-α, leading to pyroptosis [26–28]. Thus, we examined whether 8-OHdG treatment affects the mRNA levels of TNF-α, TLR2, and TLR4 in DOX-treated H9c2 cells. Exposure to DOX increased the expression of TNF-α, TLR2, and TLR4 by 690–1369%, 565–937%, and 825–1264% compared to control, respectively, whereas additional treatment with 8-OHdG reduced their expression levels by 42.2–78.3%, 12.8–74%, and 39.8–75.7% compared to DOX treated groups, respectively (Fig. 4A).

To evaluate whether 8-OHdG decreased NOX1/2/4 and TLR2/4 similar to Rac1 siRNA, H9c2 were transfected with siRNAs for negative control and two kinds of rat Rac1, and then treated with DOX and/or 8-OHdG for 24 h. The inhibitory effects of 8-OHdG on the regulation of NOX1/2/4, and TLR2/4 in DOX-exposed H9c2 cells were similar to that of two kinds of Rac1 siRNA (Suppl. Fig. 1).

Next, to elucidate whether 8-OHdG mitigates DOX-induced inflammasome activation, we examined the effect of 8-OHdG on the expression of the inflammasome components, NLRP3, ASC, and caspase-1 in DOX-treated H9c2 cells.

Induced NLRP3 expression (55–70%) by DOX was reduced by 54.8–91.2% in additional treatment of 8-OHdG (Fig. 4A). In addition, DOX increased the protein levels of ASC and caspase-1 compared to those in the control group, whereas 8-OHdG treatment decreased ASC and active caspase-1 levels compared to those in DOX-treated cells (Fig. 4B). Collectively, these findings suggest that 8-OHdG may be effective in suppressing DOX-induced inflammasome activation in cardiomyocytes.

### Exogenous 8-OHdG decreases IL-1β expression, GSDMD-NT production, and pyroptosis in DOX-treated H9c2 cells

**Fig. 5:**
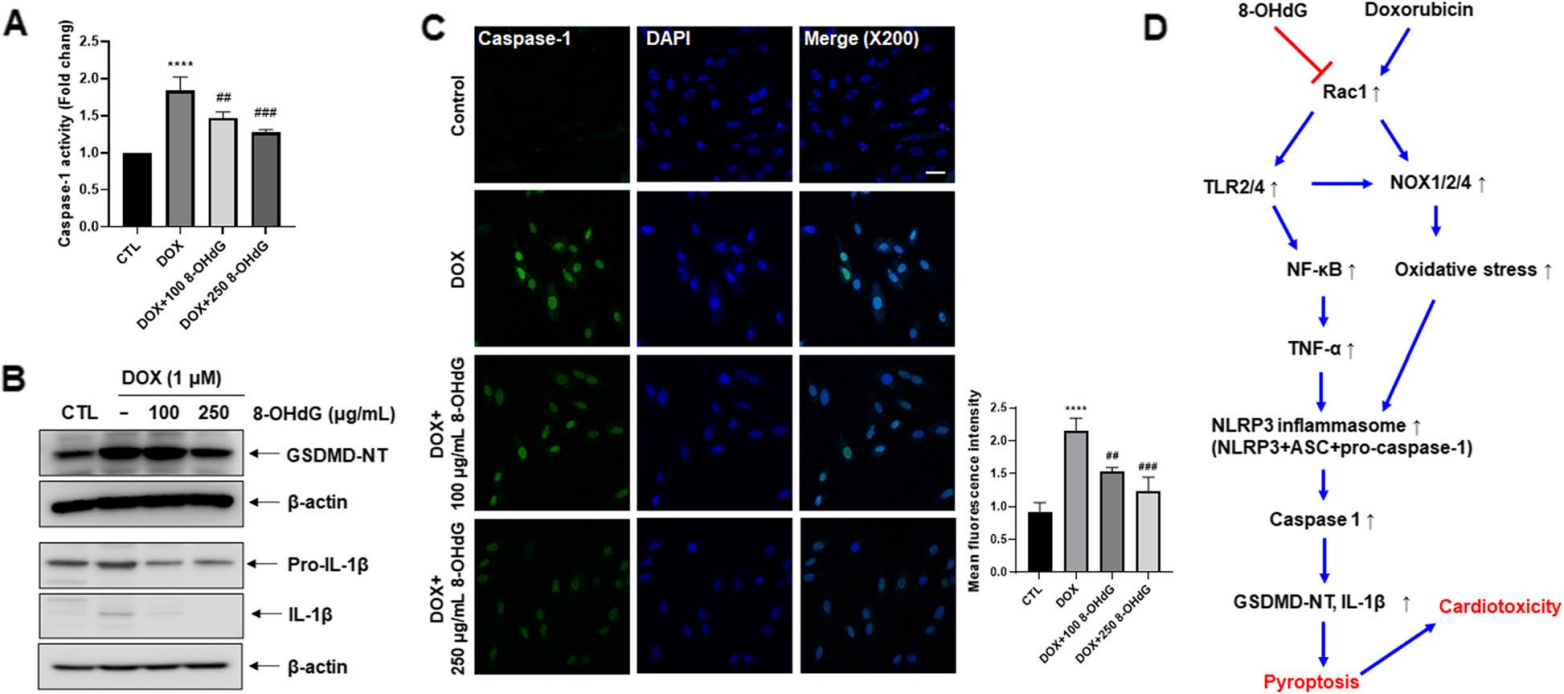
Exogenous 8-OHdG decreases IL-1β expression, GSDMD-NT levels, and pyroptosis in DOX-treated H9c2 cells. **A**–**C** H9c2 cells were treated with 1 μM DOX and 100 μg/mL or 250 μg/mL 8-OHdG for 24 h. **A** Caspase-1 activity in cells as determined by a pyroptosis/caspase-1 assay kit. Levels of active caspase-1 in the cells after treatment with DOX and 8-OHdG. After treatment with 1 μM DOX and 100 or 250 μg/mL 8-OHdG, cells were stained with FAM-YVAD-FMK, and caspase-1 activity was measured with a fluorescence microplate reader. **B** Western blot analysis of GSDMD, pro-IL-1β, IL-1β, and β-actin protein levels in DOX- and 8-OHdG-treated H9c2 cells. **C** 8-OHdG inhibited DOX-induced caspase-1 activity in H9c2 cells. Scale bar, 20 μm. After fixation, cells stained with FAM-YVAD-FMK were observed with a confocal microscope (left). Bar graph showing the quantification of active caspase-1 positive cells. 8-OHdG treatment decreased caspase-1 activity in DOX-treated cells (right). **D** Schematic diagram of the proposed mechanism for the effect of 8-OHdG in DOX-exposed cardiomyocytes. Exogenous 8-OHdG decreases the expression of NOX1/2/4, TLR2/4, and NF-κB in DOX-treated cells, which eventually leads to reduced NLRP3 inflammasome, IL-1β, and GSDMD-NT levels. Furthermore, the decrease in the levels of pyroptosis-related factors leads to the decreased expression of myocarditis-related markers, such as ANP and BNP. CTL, control; DOX, doxorubicin; 8-OHdG, 8-hydroxydeoxyguanosine; GSDMD-NT, gasdermin D N-terminal cleavage product; IL, interleukin; TLR, toll-like receptor; NOX, NADPH oxidase; NF-κB, nuclear factor kappa-light-chain-enhancer of activated B cells; TNF, tumor necrosis factor; NLRP, NLR family pyrin domain containing. ^****^P < 0.0001 versus control; ^##^*P* < 0.01 and ^###^*P* < 0.001 versus DOX-treated groups

Given that activated caspase-1 cleaves GSDMD, leading to the release of GSDMD-NT [15–17], we investigated the effect of 8-OHdG on caspase-1 activation and GSDMD cleavage in DOX-treated cells. DOX increased caspase-1 activity by 63–94.8% compared to that in the control group, and additional treatment with 8-OHdG decreased the activity by 7–36.9% compared to that in DOX-treated cells (Fig. 5A). Furthermore, 8-OHdG repressed DOX-induced GSDMD-NT production and pro-IL-1β/IL-1β expression (Fig. 5B). To confirm the inhibitory effects of 8-OHdG on DOX-induced caspase-1 activation, we observed active caspase-1 positive cells in DOX-treated H9c2 cells using confocal microscopy. As shown in Fig. 5C, DOX increased caspase-1 activity by 83.3–190% compared to that in control cells, whereas 8-OHdG decreased caspase-1 activity by 19.2–52.8% compared to that in DOX-treated H9c2 cells.

Collectively, our results suggest that DOX-induced caspase-1 activation cleaves GSDMD, which initiates pyroptosis in H9c2 cells, whereas 8-OHdG alleviates DOX-induced pyroptosis in cardiomyocytes.

## Discussion

Cardiotoxicity is the most serious complication after chemotherapy, which leads to a reduction in left ventricular ejection fraction (LVEF) of more than 10% to 50% [42]. DOX-induced cardiotoxicity is dose-dependent and occurs in 3 to 18% of patients [43]. Furthermore, DOX treatment leads to congestive heart failure, which is the most severe form of cardiotoxicity, in around 5% of patients [44].

The prognosis of DOX-induced congestive heart failure is very poor [45]. To prevent DOX-induced cardiotoxicity, regular monitoring of LVEF is recommended, and cessation of chemotherapy is also recommended when LVEF drops below 40% [46]. Currently, prophylactic or curative drugs for treating DOX-induced cardiotoxicity are sparse [47, 48]. Several standard heart failure medications including renin-angiotensin system blockers or beta blockers have been used; however, they are not very effective in the treatment or prevention of DOX-induced cardiotoxicity [47, 48]. Therefore, other targeted cardioprotective therapies or prophylactic treatments for DOX-induced cardiotoxicity should be developed [49].

The NLRP3 inflammasome has been shown to be involved in DOX-induced cardiotoxicity. DOX treatment increases NLRP3 inflammasome and IL-1β secretion in the myocardium of mice [50]. Increased pyroptosis is also involved in DOX-induced cardiotoxicity in both in vitro and in vivo models [51]. Moreover, the inhibition of the NLRP3 inflammatory response leads to decreased DOX-induced cardiotoxicity [52]. Thus, decreasing NLRP3 inflammasome or pyroptosis could be promising therapeutic methods to prevent or treat DOX-induced cardiotoxicity [53].

Increased ROS or NOX activity is known to increase NLRP3 inflammasome [34, 35]. Exogenous 8-OHdG leads to decreasing production of ROS and NOX activity via Rac1 downregulation [39]. Thus, we evaluated whether exogenous 8-OHdG decreases NLRP3 inflammasome-related inflammation and pyroptosis in DOX-treated cardiomyocytes by Rac1 inhibition. H9c2 cells are subclonal line which derived from embryonic rat heart tissue and have similarities with differentiated adult cardiac cells [54, 55], but H9c2 cells do not have ability of contraction [55]. However, H9c2 is most extensively used during preclinical studies of anticancer drug development such as evaluation of cardiotoxicity and safety [56]. Particularly, DOX-treated H9c2 model could mimic relevant mechanisms of DOX induced cardiac injuries such as oxidative stress, apoptosis, sarcoplasmic reticulum stress, and cell death [33, 57, 58]. Thus, we thought that H9c2 would be proper cell line to evaluated DOX-induced pyroptosis.

In our study, 8-OHdG decreased Rac1 activity in DOX-treated H9c2 cells (Fig. 2A). Previous study showed that DOX induced Rac1 medicated NOX activation [59]. NOX1/2/4 expression was significantly increased by DOX in H9c2 cells, and was significantly decreased by additional treatment with 8-OHdG. Furthermore, we evaluated oxidative stress by measuring the GSH/GSSG ratio and found that, while DOX treatment increased oxidative stress, 8-OHdG decreased it.

Previous studies have shown that the expression of TLR2, TLR4, TNF-α, and NF-κB is increased in DOX-induced cardiotoxicity, and that the upregulation of those signals leads to an increase in NLRP3 inflammasome and pyroptosis [29–32]. We found that DOX treatment increased the expression of TLR2, TLR4, and NF-κB (p65), whereas 8-OHdG treatment decreased it. We confirmed that 8-OHdG had a similar effect to that of Rac1 siRNA in inhibition of NOX1/2/4 and TLR2/4 expression (Suppl. Fig. 1). Thus, it seemed that 8-OHdG effects on decreasing NOX1/2/4 and TLR2/4 expression were mediated by decreasing Rac1 activity.

Generation of NLRP3 inflammasome proceeds in two phases of priming and activation. During priming phase, increased transcription of NLRP3 and pro-IL-1β and pro-IL-18 upon stimulation of DAMPs or pro-inflammatory cytokines TNF-α and IL-1β [11–17]. Various signals such as ROS induce activation of NLRP3 inflammasome. NLRP3 oligomer forms inflammasome complex with ASC (an adaptor) and pro-caspase-1 (an effector) which lead to activation of caspase-1 [11–17]. Activated caspase-1 leads to cleavage of pro-IL-1β, pro-IL-18, and GSDMD into IL-1β, IL-18, and GSDMD-NT, respectively [11–17]. GSDMD-NT forms membrane pores which lead to pyroptosis and extracellular spilling of IL-1β and IL-18 which aggravates inflammation [11–17].

In our study, DOX treatment increased the expression of NLRP3 inflammasome components (NLRP3, ASC, and pro-caspase-1) and caspase-1, whereas 8-OHdG treatment decreased it. DOX treatment led to increased expression of both pro- IL-1β and IL-1β in H9c2 cells. GSDMD-NT levels were increased by DOX treatment and decreased by 8-OHdG treatment. Moreover, the amount of pyroptotic cells as evaluated by a caspase-1 activity assay was increased by DOX treatment and decreased by 8-OHdG treatment.

Both ANP and BNP are known to be useful predictors and prognostic markers of heart failure [60–62]. ANP is secreted from atria when the atrium is dilated [63–65]. BNP is synthesized in the ventricle depending on end diastolic pressure and volume, and is therefore a more sensitive marker for heart failure than ANP [63–65]. Moreover, BNP production starts to increase from week 6 to 12 of DOX-induced cardiotoxicity [65]. Several studies have shown that BNP can be used as a marker of DOX-induced cardiotoxicity [61, 62]. Both BNP and LVEF are effective predictors of hospitalization with heart failure after chemotherapy with DOX [66]. However, BNP predicts overall death after DOX-induced cardiotoxicity more accurately than LVEF [66]. On the other hand, GATA4 is a member of the GATA family of proteins, and is essential for the adaptive response of cardiomyocytes [67–69]. DOX treatment leads to GATA4 depletion, and decreased GATA4 levels are associated with DOX-induced cardiotoxicity. A 50% reduction in GATA4 levels results in a hyper-response to DOX in mice, and results in higher myocyte loss than in wild-type mice [70]. GATA6 levels are also decreased by DOX. Furthermore, the expression of GATA4/6 is decreased in DOX-treated H9c2 cells [71].

In our study, the expression of ANP and BNP was increased by DOX treatment and decreased by 8-OHdG treatment. However, the expression of GATA4/6 was decreased by DOX treatment, and 8-OHdG treatment only increased GATA6 expression. Thus, it seems that 8-OHdG decreases the expression of cardiotoxicity makers in DOX-treated H9c2 cells which was accompanied with decreased expression of TLR2/4, NF-κB, and NOX 1/2/4 and pyroptosis. DOX-treated H9c2 cells also exhibited increased expression of IL-6 which induced cell injuries as well as IL-1β or TNF-α [72]. Since exogenous 8-OHdG also leads to decrease IL-1β, TNF-α, and IL-6 [39], it is possible that 8-OHdG could the decrease expression of cardiotoxicity makers by directly reducing IL-6, IL-1β, and TNF-α as well as decreasing pyroptosis.

Immune checkpoint inhibitors (ICIs) which show effect of anti-PD-1 (nivolumab and pembrolizumab), anti-PD-L1 (atezolizumab, avelumab, and durvalumab), and anti-CTLA-4 antibodies (ipilimumab and tremelimumab) [73–75] also result in side effect of myocarditis [76]. Those ICIs lead to increased NLRP3 and MyD88 in the microcytes [77]. Thus, there is a possibility that 8-OHdG could decrease ICIs induced myocarditis by inhibiting NRLP3. We will evaluate whether 8-OHdG could decrease ICIs induced myocarditis as a future study. Ferroptosis is a non-apoptotic cell death which induced by excessive lipid peroxidation via iron-dependent activation of lipoxygenase [78, 79]. Moreover, ferroptosis is one of pathophysiology of DOX-induced cardiotoxicity [79]. DOX induces increased iron mediated ferroptosis by upregulation of NOX4 signaling [80]. Thus, we will evaluate whether 8-OHdG could decrease DOX-induced cardiotoxicity by decreasing ferroptosis as a future study.

## Conclusions

Collectively, exogenous 8-OHdG decreased the expression of NOX1/2/4, TLR2/4, and NF-κB by decreasing Rac1 activity in DOX-treated cells, which eventually led to reduced NLRP3 inflammasome, IL-1β, and GSDMD-NT levels. Furthermore, the decrease in the levels of pyroptosis-related factors led to the decreased expression of myocarditis-related markers such as ANP and BNP (Fig. 5D). Thus, exogenous 8-OHdG might potentially be used to attenuate DOX-induced cardiotoxicity through the inhibition of pyroptosis.

## Supplementary Information

**Additional file 1.**

## Data Availability

All data generated or analyzed during this study are included in this published article and its supplementary information files.
