# Supplementary material for: Exogenous 8-hydroxydeoxyguanosine attenuates doxorubicin-induced cardiotoxicity by decreasing pyroptosis in H9c2 cardiomyocytes
Source: BMC Mol Cell Biol. 2022 Dec 14;23:55. doi: 10.1186/s12860-022-00454-1 (PMC9753270; doi:10.1186/s12860-022-00454-1)
Supplement: Supplementary file 1 — Additional file 1. [file 12860_2022_454_MOESM1_ESM.zip › Supplemental Figures_BMC MCB.docx]

Exogenous 8-hydroxydeoxyguanosine attenuates doxorubicin-induced cardiotoxicity by decreasing pyroptosis in H9c2 cardiomyocytes

Soyoung Hwang ^a, 1^, Se-Hee Kim ^b, 1^, Kwai Han Yoo ^c, 1^, Myung-Hee Chung ^d^,

Jin Woo Lee ^e, f, *^, and Kuk Hui Son ^a, *^

^a^ *Department of Thoracic and Cardiovascular Surgery, Gachon University Gil Medical Center, College of Medicine, Gachon University, 21, Namdong-daero 774 beon-gil,*

*Namdong-gu, Incheon, Republic of Korea*; [snrntlwy1004@gmail.com](mailto:snrntlwy1004@gmail.com) (S.H.);

[dr632@gilhospital.com](mailto:dr632@gilhospital.com) (K.H.S.)

^b^ *Gachon Medical Research Institute, Gachon University Gil Medical Center, College of*

*Medicine, Gachon University, 21, Namdong-daero 774 beon-gil, Namdong-gu, Incheon,*

*Republic of Korea*; [sehee0423@gilhospital.com](mailto:sehee0423@gilhospital.com) (S.-H.K.)

^c^ *Division of Hematology, Department of Internal Medicine, Gachon University Gil Medical Center, Gachon University College of Medicine, Incheon, Korea;*

[khyoo@gilhospital.com](mailto:khyoo@gilhospital.com) (K.H.Y)

^d^ *Lee Gil Ya Cancer and Diabetes Institute, Gachon University, 155, Gaetbeol-ro,*

*Yeonsu-ku, Incheon, Republic of Korea*; [mhchung@snu.ac.kr](mailto:mhchung@snu.ac.kr) (M.-H.C.)

^e^ *Department of Molecular Medicine, College of Medicine, Gachon University, 155,*

*Gaetbeol-ro, Yeonsu-ku, Incheon, Republic of Korea*; [jwlee@gachon.ac.kr](mailto:jwlee@gachon.ac.kr) (J.W.L.)

^f^ *Department of Health Sciences and Technology, GAIHST, Gachon University, 155,*

*Gaetbeol-ro, Yeonsu-ku, Incheon, Republic of Korea*; [jwlee@gachon.ac.kr](mailto:jwlee@gachon.ac.kr)

^1^ Contributed equally

***** Correspondence: [dr632@gilhospital.com](mailto:dr632@gilhospital.com) (K.H.S.) and [jwlee@gachon.ac.kr](mailto:jwlee@gachon.ac.kr) (J.W.L.)

**Supplemental figure titles and legends**


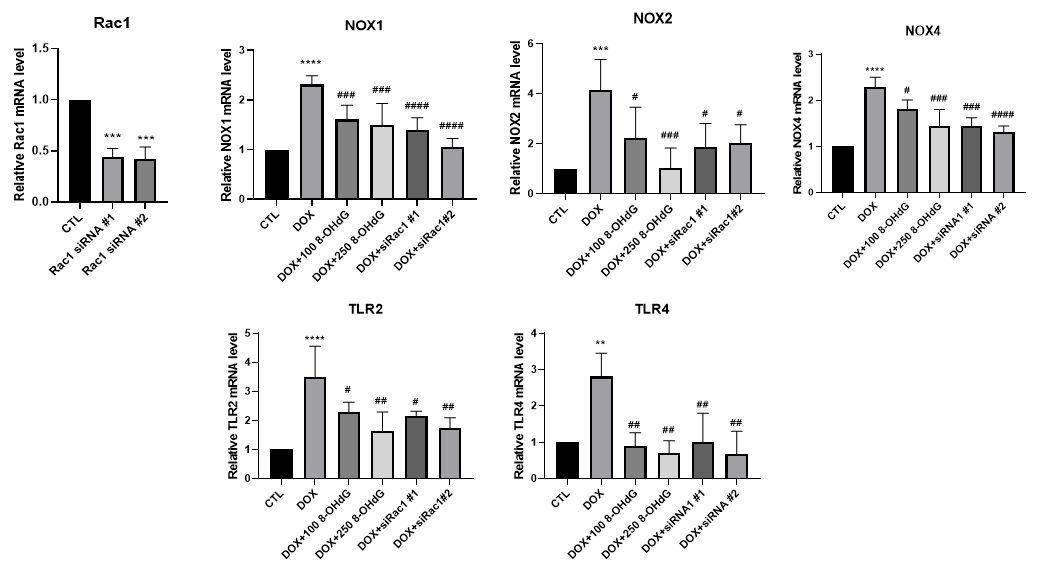


**Suppl. Fig. 1. Exogenous 8-OHdG decreases NOX1/2/4 and TLR2/4 expression in DOX-treated H9c2 cells.** After cells were transfected with siRNAs for negative control or Rac1 (#1 or #2), H9c2 cells were treated with 1 μM DOX and 100 or 250 μg/mL 8-OHdG for 24 h. Quantitative real-time PCR for NOX1/2/4 and TLR2/4 were performed. CTL, control; DOX, doxorubicin; 8-OHdG, 8-hydroxydeoxyguanosine. ^**^*P* < 0.01, ^***^*P* < 0.001, and ^****^*P* < 0.0001 versus CTL; ^#^*P* < 0.05, ^##^*P* < 0.01, ^###^*P* < 0.001, and ^####^*P* < 0.0001 versus DOX.

**Supplemental Methods**

*RNA interference transfection.* Cells (3X10^5^/60mm dish) were transfected with 40 nM siRNAs (Bioneer, Daejeon, South Korea) for negative control (Bioneer) and rat Rac1 (#1 and #2) using Lipofectamine 2000 (Invitrogen, Carlsbad, CA, USA), and then were treated with DOX (1 μM) and 8-OHdG (100 or 250 μg/mL) for 24 h. Sequences for siRNAs were as follows: rat Rac1 #1 (5'-AGC CGG CAA AGG GA-3') and rat Rac1 #2 (5'-GGG AGA AAG GAC ACG A-3').

*Quantitative reverse transcription PCR (qRT-PCR).* Total RNA was isolated from H9c2 cells using RNAiso plus (Takara Bio Inc., Otsu, Shiga, Japan). RNA quantitation was performed using Nano drop-2000 (Thermo Fisher Scientific, Waltham, MA, USA). Then, cDNA synthesis was performed using a PrimeScript First Strand cDNA Synthesis Kit (Takara Bio). Real-time PCR was performed on a CFX 96 real-time system (Bio-Rad, Hercules, CA, USA) using SYBR Green I Universal PCR Master Mix (Takara Bio) and primers (Bioneer) in reactions with the following conditions: 95°C for 10 min, followed by 40 cycles of 95°C for 15 s, and 60°C for 1 min. GAPDH was used as a reference gene. Relative gene expression presents the data of the gene of interest relative to internal control gene using the comparative *C*_T_ method also referred to as the 2^-△△^*^C^*^T^ method. Primer (Bioneer, Daejeon, South Korea) sequences for Rac1, NOX1, NOX2, NOX4, TLR-2, TLR-4, and GAPDH were as follows: Rac1-F, 5’-AGGTGTCTGCATGGTGGTG-3’ and Rac1-R, 5’-GAGGGTGAGTGTCTAAATTGGT-3’; NOX1-F, 5’-TCCTAAACTACCGACTCTTC-3’ and NOX1-R, 5’-GTCCCACATTGGTCTCCC-3’; NOX2-F, 5’-TCAAGTGTCCCCAGGTATCC-3’ and NOX2-R, 5’-CTTCACTGGCTGTACCAAAGG-3’; NOX4-F, 5’- AGGTGTCTGCATGGTGGTG-3’ and NOX4-R, 5’- GAGGGTGAGTGTCTAAATTGGT -3’; TLR2-F, 5’-GGCCACAGGACTCAAGAGCA-3’ and TLR2-R, 5’-AGAGGCCTATCACAGCCATCAAG-3’, TLR-4F, 5’-GGACTCTGCCCTGCCACCATTTA-3’; and TLR4-R 5’-CTTGTGCCCTGTGAGGTCGTTGA-3’; GAPDH-F, 5’-GGCTCTCTGCTCCTCCCTGTTCTA-3’ and GAPDH-R, 5’-TGCCGTTGAACTTGCCGTGGG-3.’
