# Supplementary material for: Exogenous 8-hydroxydeoxyguanosine attenuates doxorubicin-induced cardiotoxicity by decreasing pyroptosis in H9c2 cardiomyocytes
Source: BMC Mol Cell Biol. 2022 Dec 14;23:55. doi: 10.1186/s12860-022-00454-1 (PMC9753270; doi:10.1186/s12860-022-00454-1)
Supplement: Supplementary file 1 — Additional file 1. [file 12860_2022_454_MOESM1_ESM.zip › Supplementary file_Datasets.pptx]

## Slide 1
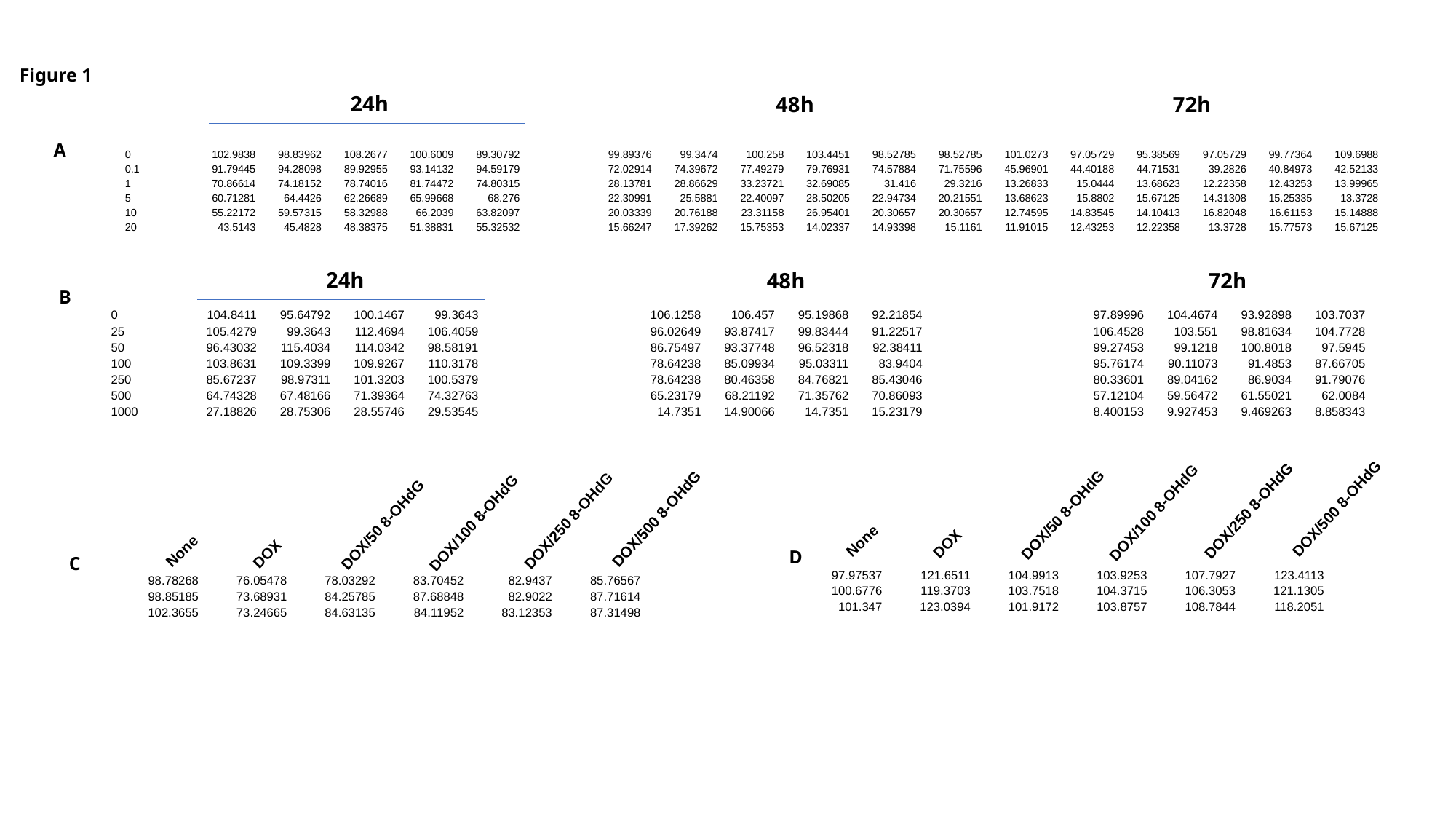

Figure 1
24h
72h
48h
A
| 0 | 102.9838 | 98.83962 | 108.2677 | 100.6009 | 89.30792 | | 99.89376 | 99.3474 | 100.258 | 103.4451 | 98.52785 | 98.52785 | 101.0273 | 97.05729 | 95.38569 | 97.05729 | 99.77364 | 109.6988 |
| --- | --- | --- | --- | --- | --- | --- | --- | --- | --- | --- | --- | --- | --- | --- | --- | --- | --- | --- |
| 0.1 | 91.79445 | 94.28098 | 89.92955 | 93.14132 | 94.59179 | | 72.02914 | 74.39672 | 77.49279 | 79.76931 | 74.57884 | 71.75596 | 45.96901 | 44.40188 | 44.71531 | 39.2826 | 40.84973 | 42.52133 |
| 1 | 70.86614 | 74.18152 | 78.74016 | 81.74472 | 74.80315 | | 28.13781 | 28.86629 | 33.23721 | 32.69085 | 31.416 | 29.3216 | 13.26833 | 15.0444 | 13.68623 | 12.22358 | 12.43253 | 13.99965 |
| 5 | 60.71281 | 64.4426 | 62.26689 | 65.99668 | 68.276 | | 22.30991 | 25.5881 | 22.40097 | 28.50205 | 22.94734 | 20.21551 | 13.68623 | 15.8802 | 15.67125 | 14.31308 | 15.25335 | 13.3728 |
| 10 | 55.22172 | 59.57315 | 58.32988 | 66.2039 | 63.82097 | | 20.03339 | 20.76188 | 23.31158 | 26.95401 | 20.30657 | 20.30657 | 12.74595 | 14.83545 | 14.10413 | 16.82048 | 16.61153 | 15.14888 |
| 20 | 43.5143 | 45.4828 | 48.38375 | 51.38831 | 55.32532 | | 15.66247 | 17.39262 | 15.75353 | 14.02337 | 14.93398 | 15.1161 | 11.91015 | 12.43253 | 12.22358 | 13.3728 | 15.77573 | 15.67125 |
24h
72h
48h
B
| 0 | 104.8411 | 95.64792 | 100.1467 | 99.3643 | | | 106.1258 | 106.457 | 95.19868 | 92.21854 | | | 97.89996 | 104.4674 | 93.92898 | 103.7037 |
| --- | --- | --- | --- | --- | --- | --- | --- | --- | --- | --- | --- | --- | --- | --- | --- | --- |
| 25 | 105.4279 | 99.3643 | 112.4694 | 106.4059 | | | 96.02649 | 93.87417 | 99.83444 | 91.22517 | | | 106.4528 | 103.551 | 98.81634 | 104.7728 |
| 50 | 96.43032 | 115.4034 | 114.0342 | 98.58191 | | | 86.75497 | 93.37748 | 96.52318 | 92.38411 | | | 99.27453 | 99.1218 | 100.8018 | 97.5945 |
| 100 | 103.8631 | 109.3399 | 109.9267 | 110.3178 | | | 78.64238 | 85.09934 | 95.03311 | 83.9404 | | | 95.76174 | 90.11073 | 91.4853 | 87.66705 |
| 250 | 85.67237 | 98.97311 | 101.3203 | 100.5379 | | | 78.64238 | 80.46358 | 84.76821 | 85.43046 | | | 80.33601 | 89.04162 | 86.9034 | 91.79076 |
| 500 | 64.74328 | 67.48166 | 71.39364 | 74.32763 | | | 65.23179 | 68.21192 | 71.35762 | 70.86093 | | | 57.12104 | 59.56472 | 61.55021 | 62.0084 |
| 1000 | 27.18826 | 28.75306 | 28.55746 | 29.53545 | | | 14.7351 | 14.90066 | 14.7351 | 15.23179 | | | 8.400153 | 9.927453 | 9.469263 | 8.858343 |
DOX/500 8-OHdG
DOX/250 8-OHdG
DOX/100 8-OHdG
DOX/50 8-OHdG
DOX/500 8-OHdG
DOX/250 8-OHdG
DOX/100 8-OHdG
DOX/50 8-OHdG
None
DOX
None
DOX
D
C
| 97.97537 | 121.6511 | 104.9913 | 103.9253 | 107.7927 | 123.4113 |
| --- | --- | --- | --- | --- | --- |
| 100.6776 | 119.3703 | 103.7518 | 104.3715 | 106.3053 | 121.1305 |
| 101.347 | 123.0394 | 101.9172 | 103.8757 | 108.7844 | 118.2051 |
| 98.78268 | 76.05478 | 78.03292 | 83.70452 | 82.9437 | 85.76567 |
| --- | --- | --- | --- | --- | --- |
| 98.85185 | 73.68931 | 84.25785 | 87.68848 | 82.9022 | 87.71614 |
| 102.3655 | 73.24665 | 84.63135 | 84.11952 | 83.12353 | 87.31498 |

## Slide 2
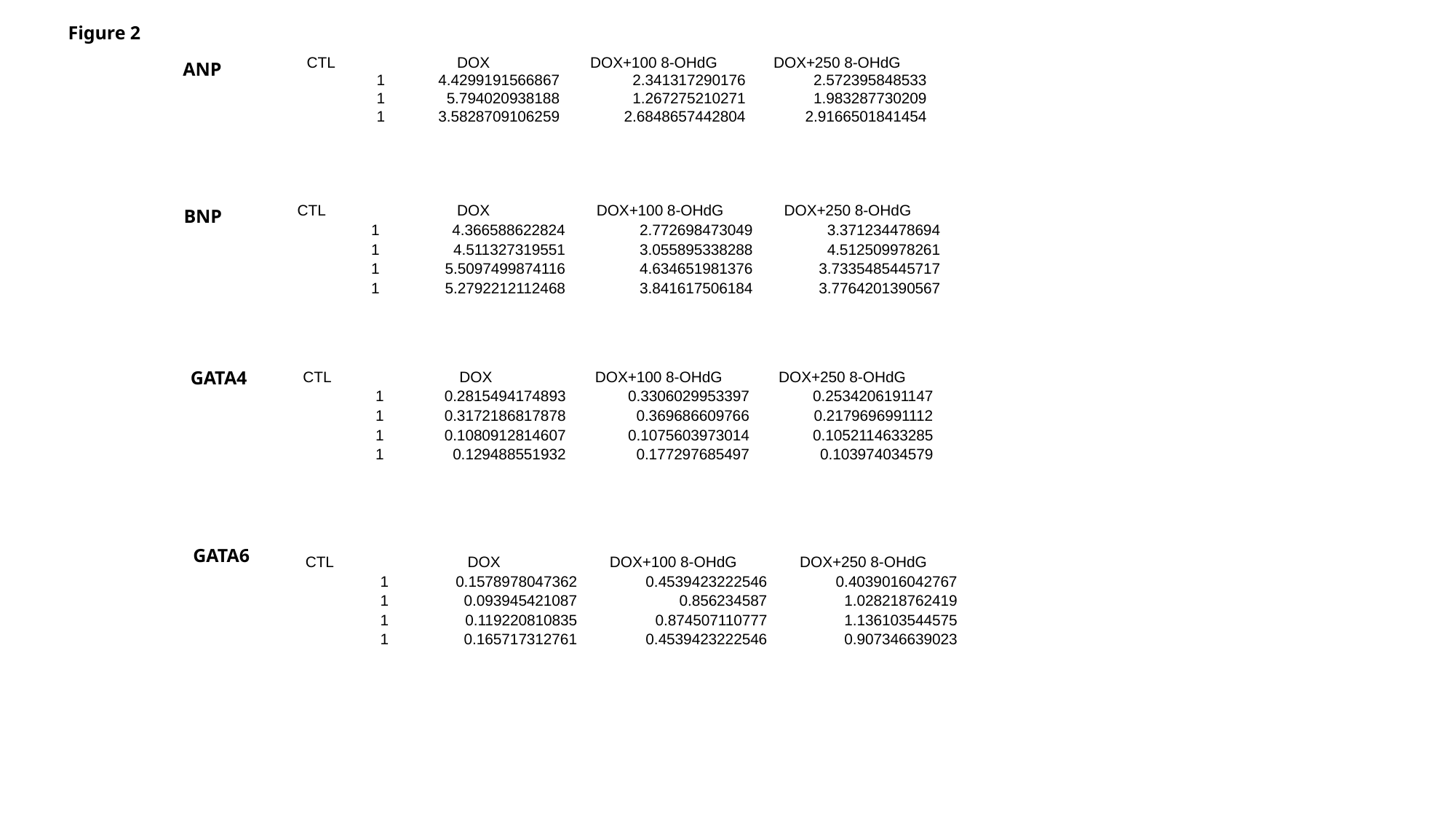

Figure 2
ANP
| CTL | DOX | DOX+100 8-OHdG | DOX+250 8-OHdG |
| --- | --- | --- | --- |
| 1 | 4.4299191566867 | 2.341317290176 | 2.572395848533 |
| 1 | 5.794020938188 | 1.267275210271 | 1.983287730209 |
| 1 | 3.5828709106259 | 2.6848657442804 | 2.9166501841454 |
BNP
| CTL | DOX | DOX+100 8-OHdG | DOX+250 8-OHdG |
| --- | --- | --- | --- |
| 1 | 4.366588622824 | 2.772698473049 | 3.371234478694 |
| 1 | 4.511327319551 | 3.055895338288 | 4.512509978261 |
| 1 | 5.5097499874116 | 4.634651981376 | 3.7335485445717 |
| 1 | 5.2792212112468 | 3.841617506184 | 3.7764201390567 |
GATA4
| CTL | DOX | DOX+100 8-OHdG | DOX+250 8-OHdG |
| --- | --- | --- | --- |
| 1 | 0.2815494174893 | 0.3306029953397 | 0.2534206191147 |
| 1 | 0.3172186817878 | 0.369686609766 | 0.2179696991112 |
| 1 | 0.1080912814607 | 0.1075603973014 | 0.1052114633285 |
| 1 | 0.129488551932 | 0.177297685497 | 0.103974034579 |
GATA6
| CTL | DOX | DOX+100 8-OHdG | DOX+250 8-OHdG |
| --- | --- | --- | --- |
| 1 | 0.1578978047362 | 0.4539423222546 | 0.4039016042767 |
| 1 | 0.093945421087 | 0.856234587 | 1.028218762419 |
| 1 | 0.119220810835 | 0.874507110777 | 1.136103544575 |
| 1 | 0.165717312761 | 0.4539423222546 | 0.907346639023 |

## Slide 3
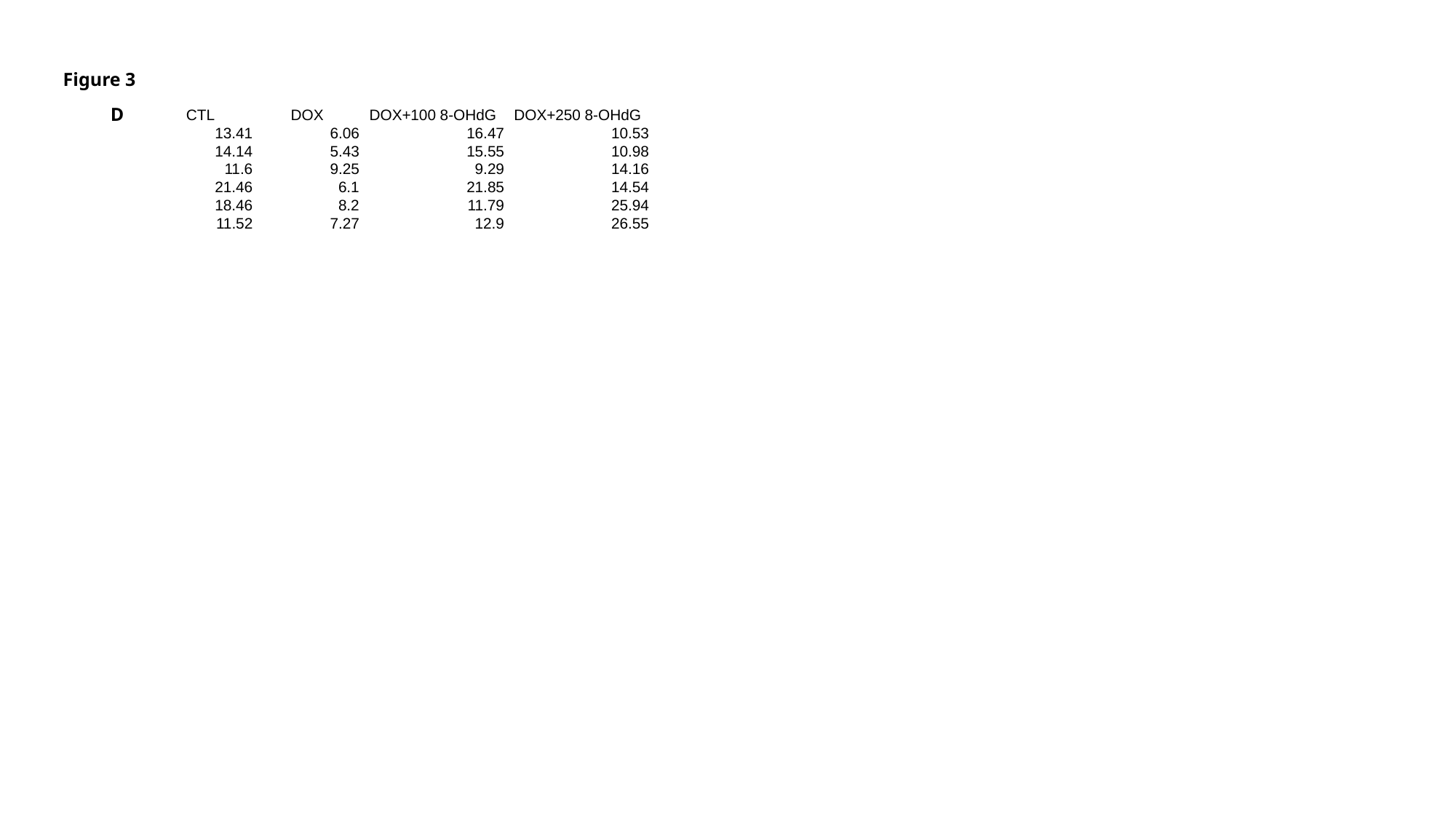

Figure 3
D
| CTL | DOX | DOX+100 8-OHdG | DOX+250 8-OHdG |
| --- | --- | --- | --- |
| 13.41 | 6.06 | 16.47 | 10.53 |
| 14.14 | 5.43 | 15.55 | 10.98 |
| 11.6 | 9.25 | 9.29 | 14.16 |
| 21.46 | 6.1 | 21.85 | 14.54 |
| 18.46 | 8.2 | 11.79 | 25.94 |
| 11.52 | 7.27 | 12.9 | 26.55 |

## Slide 4
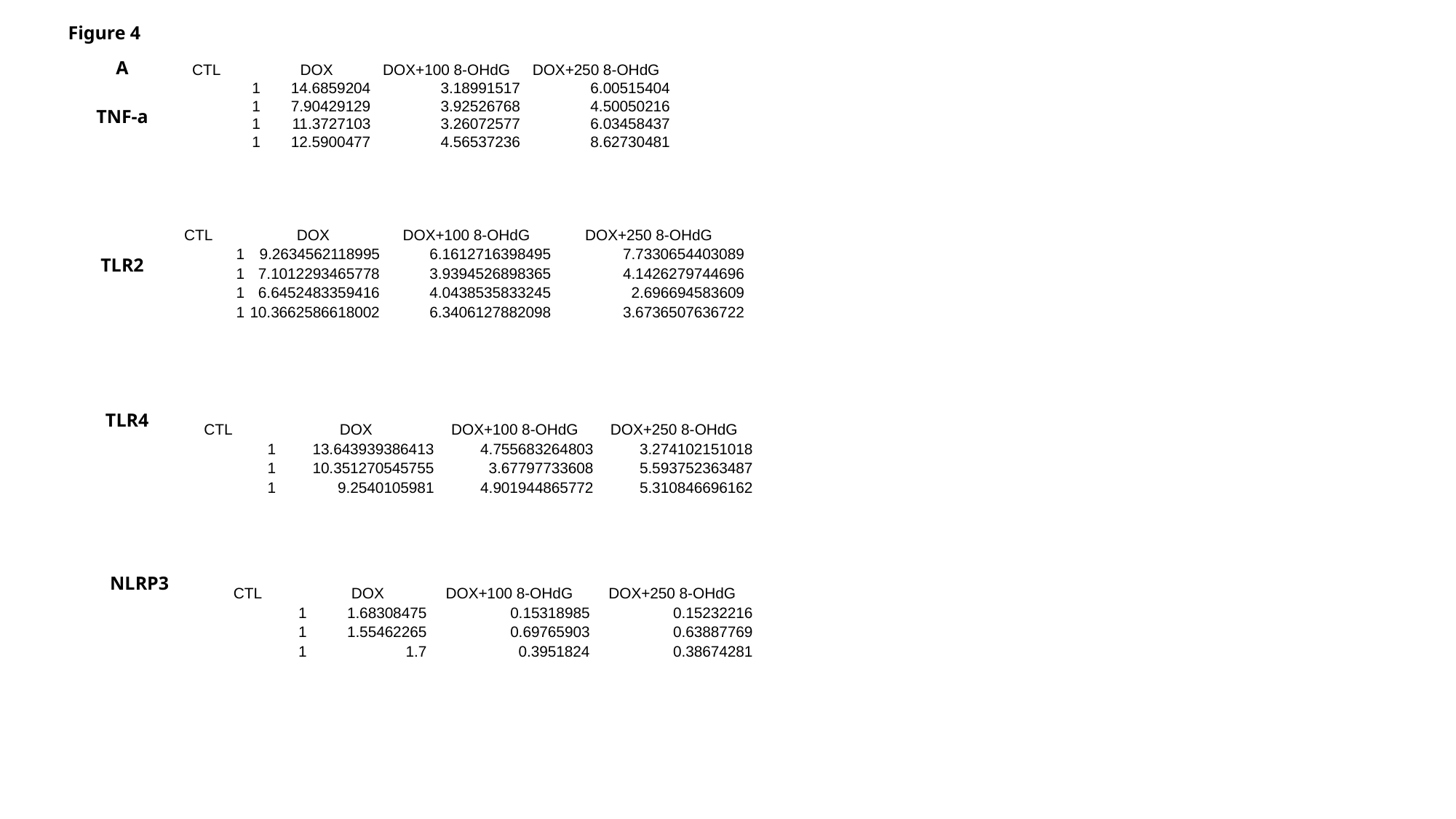

Figure 4
A
| CTL | DOX | DOX+100 8-OHdG | DOX+250 8-OHdG |
| --- | --- | --- | --- |
| 1 | 14.6859204 | 3.18991517 | 6.00515404 |
| 1 | 7.90429129 | 3.92526768 | 4.50050216 |
| 1 | 11.3727103 | 3.26072577 | 6.03458437 |
| 1 | 12.5900477 | 4.56537236 | 8.62730481 |
TNF-a
| CTL | DOX | DOX+100 8-OHdG | DOX+250 8-OHdG |
| --- | --- | --- | --- |
| 1 | 9.2634562118995 | 6.1612716398495 | 7.7330654403089 |
| 1 | 7.1012293465778 | 3.9394526898365 | 4.1426279744696 |
| 1 | 6.6452483359416 | 4.0438535833245 | 2.696694583609 |
| 1 | 10.3662586618002 | 6.3406127882098 | 3.6736507636722 |
TLR2
TLR4
| CTL | DOX | DOX+100 8-OHdG | DOX+250 8-OHdG |
| --- | --- | --- | --- |
| 1 | 13.643939386413 | 4.755683264803 | 3.274102151018 |
| 1 | 10.351270545755 | 3.67797733608 | 5.593752363487 |
| 1 | 9.2540105981 | 4.901944865772 | 5.310846696162 |
NLRP3
| CTL | DOX | DOX+100 8-OHdG | DOX+250 8-OHdG |
| --- | --- | --- | --- |
| 1 | 1.68308475 | 0.15318985 | 0.15232216 |
| 1 | 1.55462265 | 0.69765903 | 0.63887769 |
| 1 | 1.7 | 0.3951824 | 0.38674281 |

## Slide 5
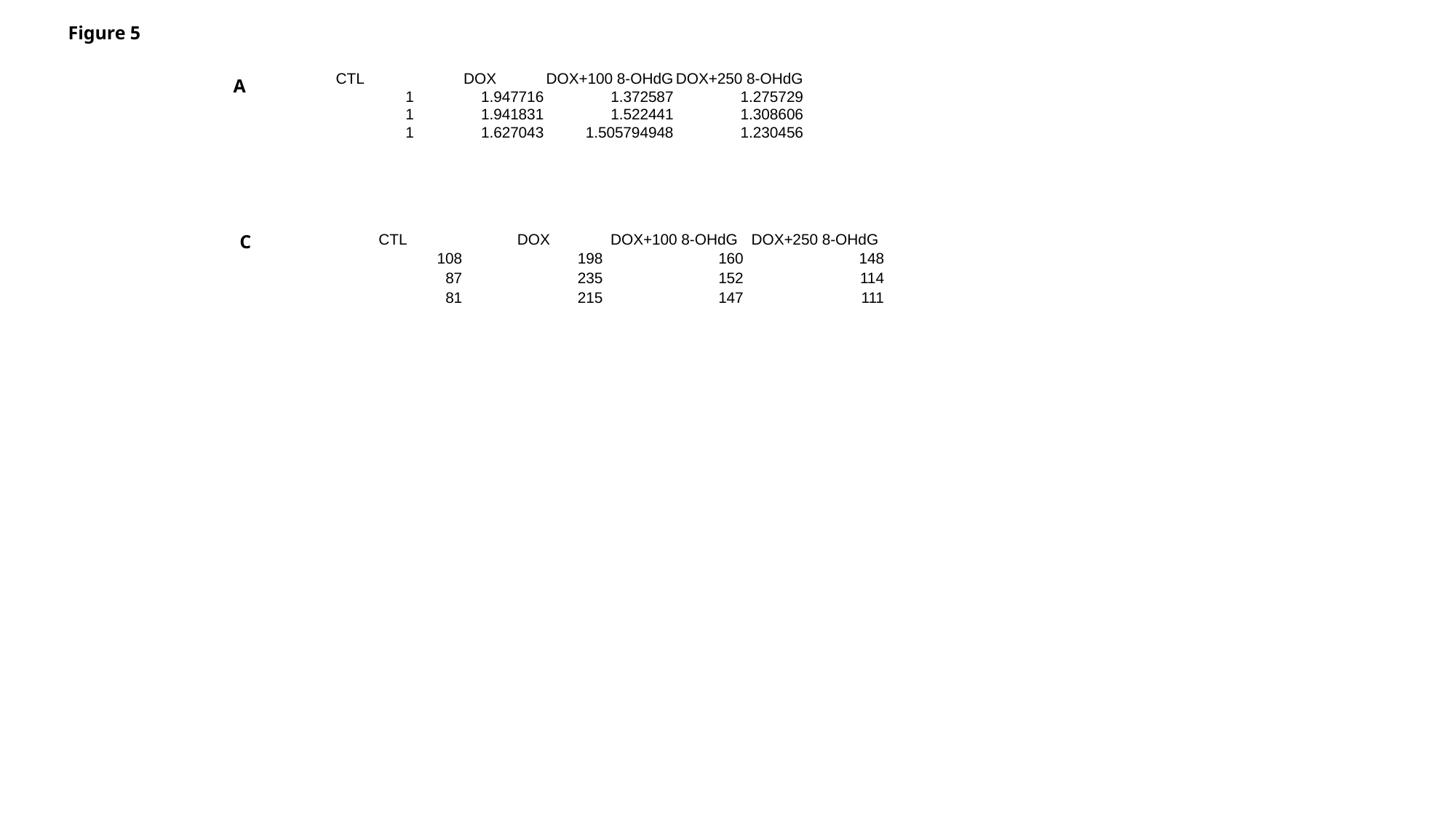

Figure 5
A
| CTL | DOX | DOX+100 8-OHdG | DOX+250 8-OHdG |
| --- | --- | --- | --- |
| 1 | 1.947716 | 1.372587 | 1.275729 |
| 1 | 1.941831 | 1.522441 | 1.308606 |
| 1 | 1.627043 | 1.505794948 | 1.230456 |
C
| CTL | DOX | DOX+100 8-OHdG | DOX+250 8-OHdG |
| --- | --- | --- | --- |
| 108 | 198 | 160 | 148 |
| 87 | 235 | 152 | 114 |
| 81 | 215 | 147 | 111 |
