# Supplementary figures and images for: Exogenous 8-hydroxydeoxyguanosine attenuates doxorubicin-induced cardiotoxicity by decreasing pyroptosis in H9c2 cardiomyocytes
Source: BMC Mol Cell Biol. 2022 Dec 14;23:55. doi: 10.1186/s12860-022-00454-1 (PMC9753270; doi:10.1186/s12860-022-00454-1)

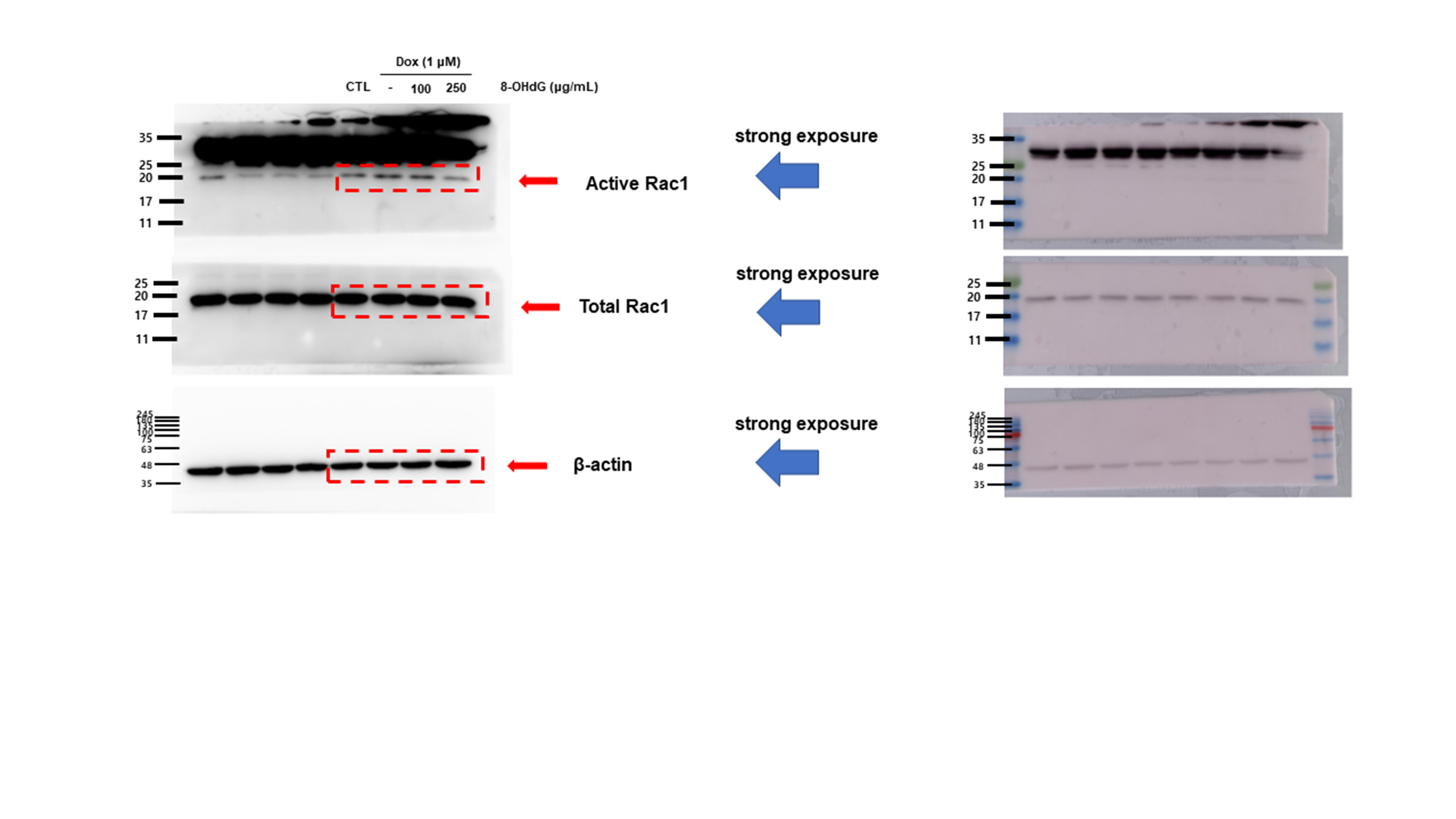

Supplement: Supplementary file 1 — Additional file 1. [file 12860_2022_454_MOESM1_ESM.zip › Figure 3A.tif]

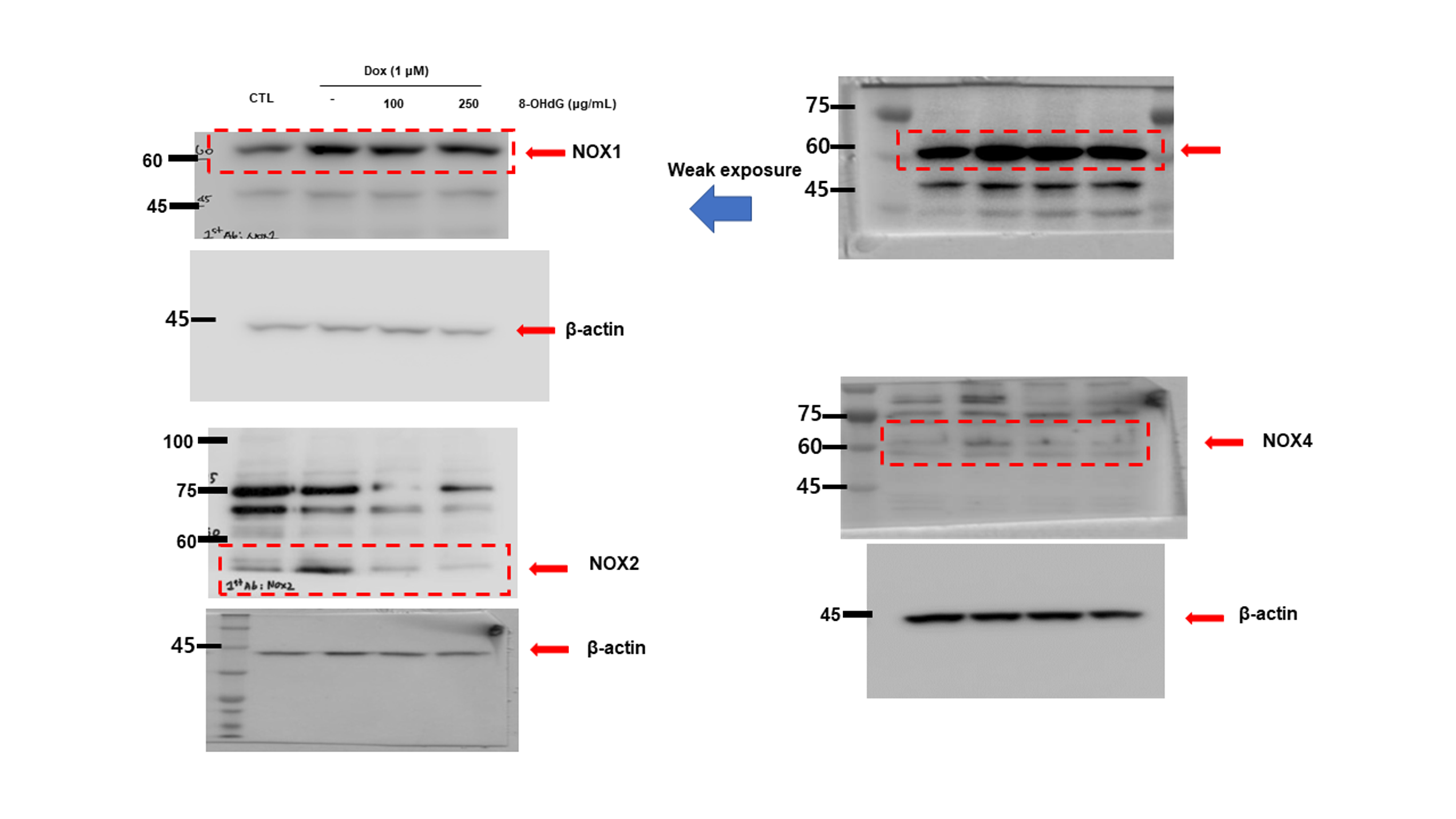

Supplement: Supplementary file 1 — Additional file 1. [file 12860_2022_454_MOESM1_ESM.zip › Figure 3B.tif]

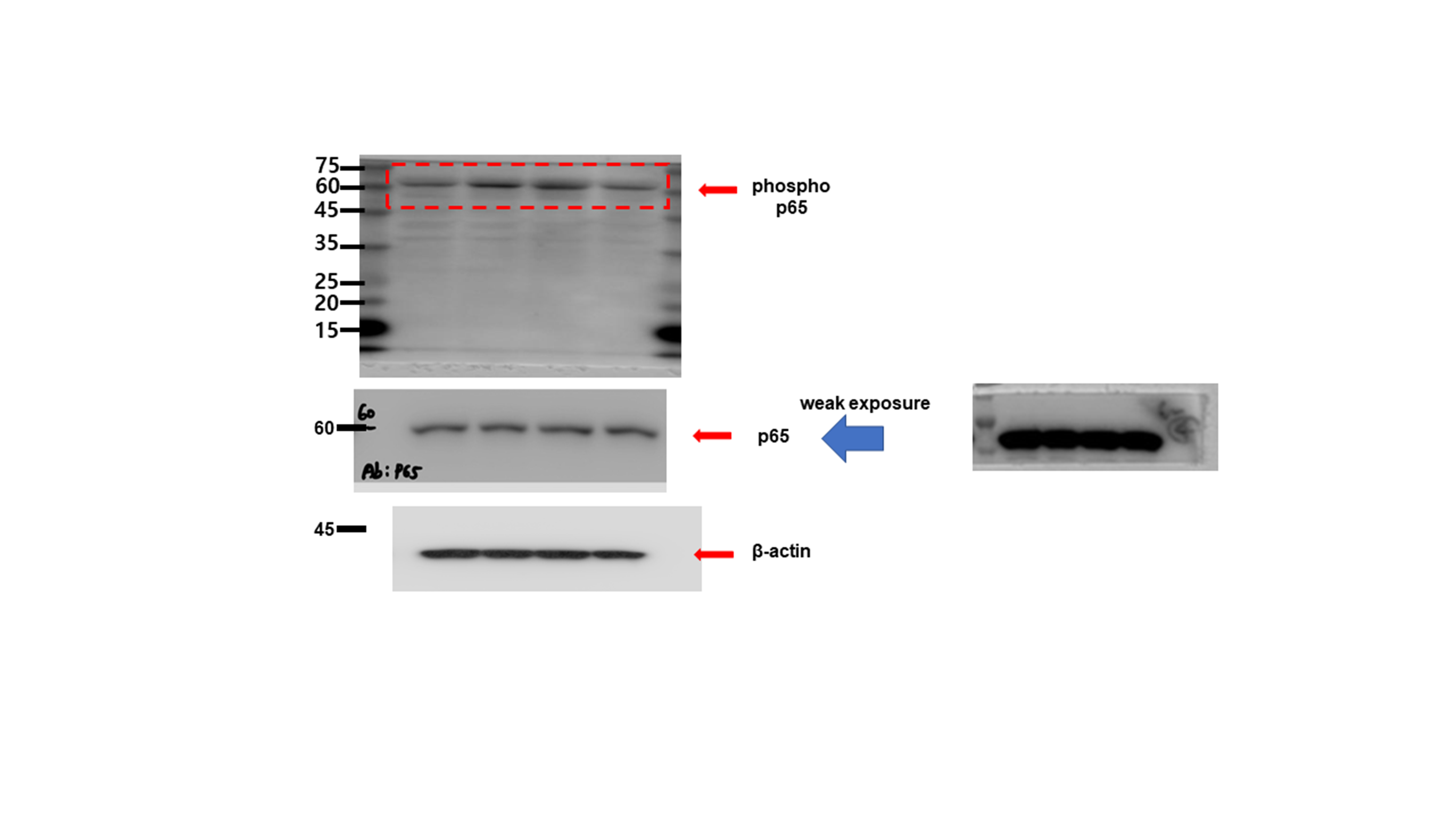

Supplement: Supplementary file 1 — Additional file 1. [file 12860_2022_454_MOESM1_ESM.zip › Figure 3C.tif]

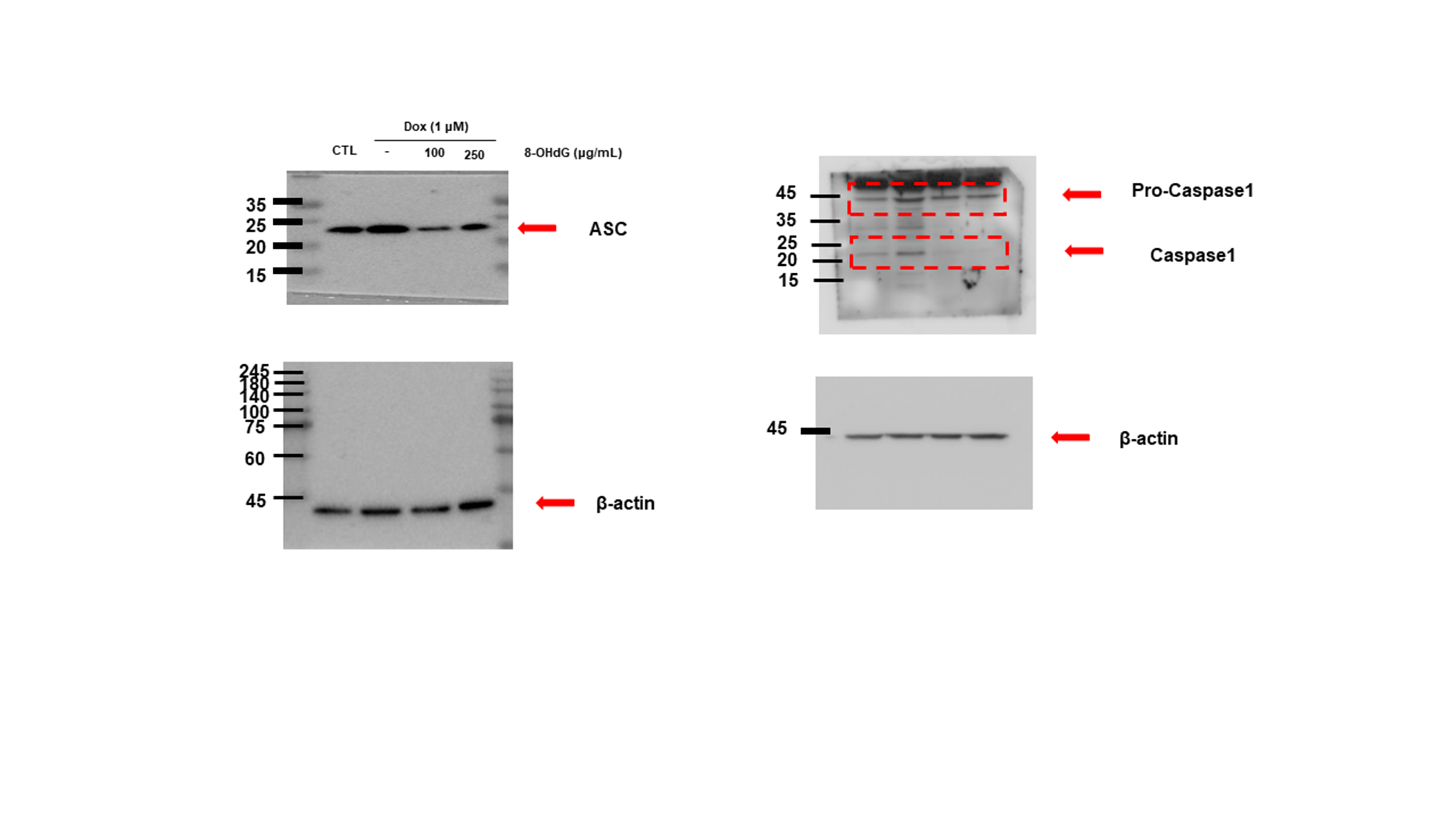

Supplement: Supplementary file 1 — Additional file 1. [file 12860_2022_454_MOESM1_ESM.zip › Figure 4B.tif]

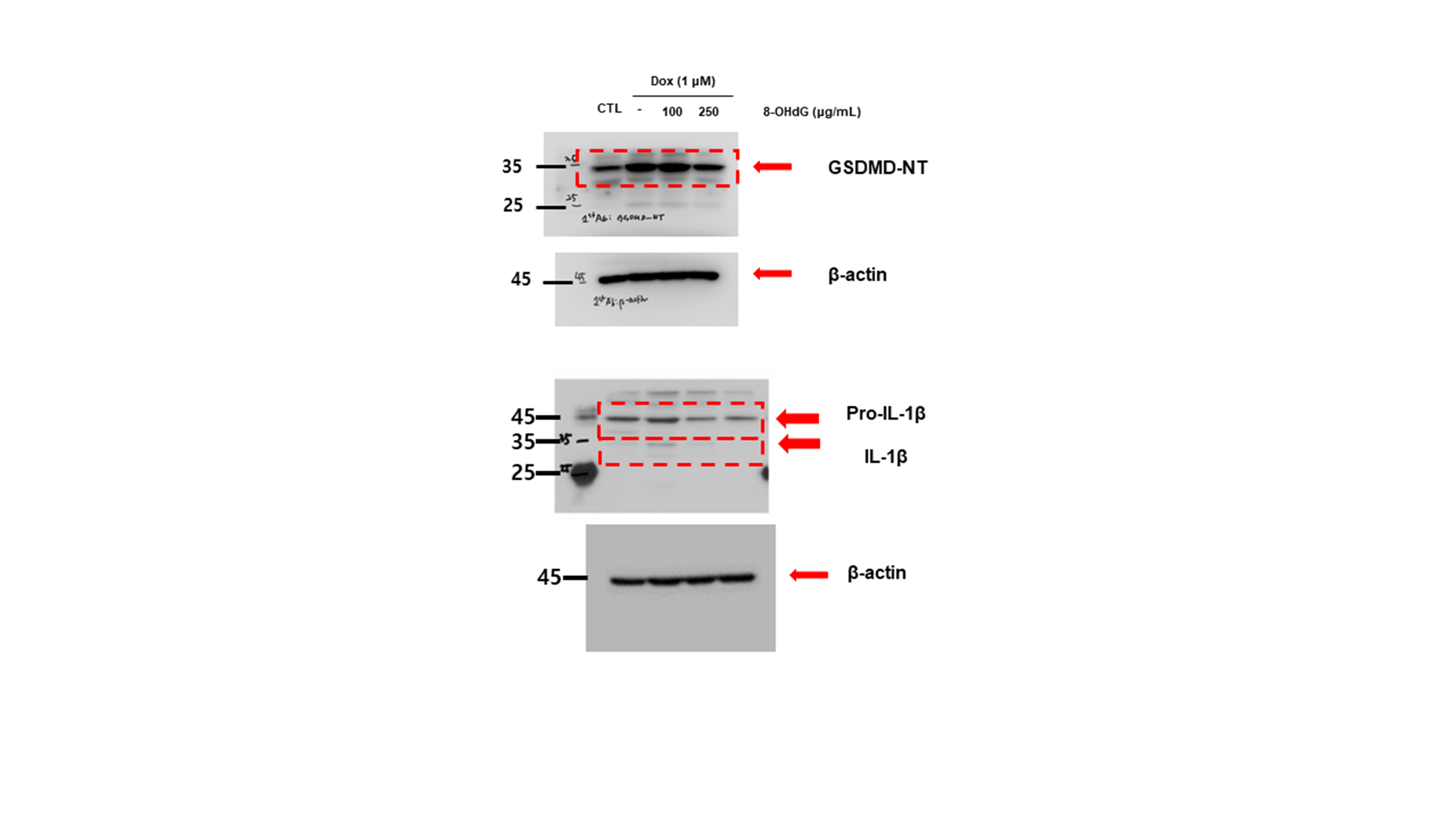

Supplement: Supplementary file 1 — Additional file 1. [file 12860_2022_454_MOESM1_ESM.zip › Figure 5B.tif]
